# Why “communicating like a woman” can work: gender stereotypes and outcomes in medical settings

**DOI:** 10.3389/fpsyg.2026.1539852

**Published:** 2026-07-16

**Authors:** Roberta Di Pasquale, Rosalba Morese

**Affiliations:** 1 University of Bergamo, Bergamo, Italy; 2 University of Italian Switzerland, Lugano, Ticino, Switzerland

**Keywords:** counter narratives for men, diagnosis, doctor-patient communication system, gender stereotypes, treatment

“Men are more determined and strong-willed”, “Women are multitaskers and capable of greater empathy”: these are just some examples of the beliefs that deeply permeate our way of thinking and reading the world. It is almost always the female sex that loses out: to a person who appears weak, we say, “don't be a sissy”, and we say to someone determined and authoritative, “you have the attributes”. Gender stereotypes concern multiple aspects of an individual's life and personality: from professional life to private life, from character attitudes to roles within the family unit, from clothing style to personal tastes. It is considered normal for a man to have a leadership role in the workplace, but it is not as obvious for a woman. It is considered natural for a woman not to work to dedicate herself to her family, but it is not considered acceptable for a man to do so. Stereotypes are harmful not only in terms of discrimination and prejudice but also in terms of limiting opportunities and life experiences, since they often prevent one from exploring one's potential.

## The female face of medicine: “the good side” of stereotypes

Healthcare workers, both men and women, are subject to the stereotypes that permeate their culture; in the case of female healthcare professionals, these stereotypes, at least in regard to the variable of doctor-patient communication, prove to be, at least for once, successful for women. The characteristics and expectations socially and culturally attributed and, in the majority of cases, embodied by women account for better doctor-patient communication, especially when the patient is a woman, which leads us to reflect on the added value of listening from a female doctor, for example, to a woman who is a victim of violence.

Today, the presence of women doctors is increasing rapidly, and they have already overtaken men in almost all specializations (Jefferson et al., 2015), surprisingly even in those fields with a traditionally male connotation, such as surgery. The “feminization” of medical professions has become an important reality throughout the Western world that is changing the face of modern medicine. Therefore, processes of prevention, diagnosis, and treatment will be affected by a new approach to healthcare, practiced and organized with a more feminine perspective. In the treatment setting, four dyads are possible: female patient-male doctor, male patient-female doctor, female patient-female doctor, and male patient-male doctor.

These dyads differ in various characteristics: communication and reception styles, administration of drugs, prescription of clinical-instrumental investigations, and more.

Focusing on communication styles, it is clear that they can directly influence clinical results, exerting a positive influence not only on the patient's health from an emotional point of view, but also on the resolution of symptoms and on the functional and physiological state. In other words, communication can also directly affect the prognosis, since it mainly performs three important functions: transmission of information, recognition of the patient's emotions, and achievement of diagnostic-therapeutic objectives. Female doctors use greater modulation of verbal communication patterns, adopt an empathetic and supportive approach, and dedicate extensive attention to the patient's emotions. Female doctors offer emotional support, encouragement, and reassurance, using a softer, friendlier tone of voice and nonverbal body language. They make more use of physical contact to reduce interpersonal distance. Furthermore, their behavior shows a greater ability to listen, expressed through simple gestures such as nodding and eye contact, which denote a certain interest in the conversation and listening to the patient. Female doctors adopt a communication style that involves sharing care processes with the patient and is based on listening, human warmth, and a desire to help. In this regard, Tannen, in one of his studies, defines the male communication style as “report talk”, based on the exchange of medical-scientific information, and the female one as “rapport talk,” based on the construction of a bilateral relationship that aims to establish sharing of the treatment process (Tannen, 1990).

Women doctors seem to establish caring relationships based on encouragement and “lowered dominance”, focused on the patient's health needs. Women doctors, in fact, obtain better results (Cousin et al., 2012) than their male colleagues, who are characterized by a “high dominance approach”, devoid of sharing, in which the patient is inhibited, speaks less, and provides less anamnestic information, with the risk of compromising treatment adherence. This is an issue with both female and male patients, but it is more prevalent with male patients. Both female and male doctors struggle to relate to male patients, above all because, unlike female patients, consistent with gender roles, they are less familiar with clinics and healthcare facilities and less likely to “ask for help”. These are all aspects that traditional gender ideology has emphasized as typical of the feminine in a negative sense. These same aspects are certainly negative if they force women into a position of subordination or are used to maintain a presumed male superiority, but they can, however, become an added value when used as a unique female competence, refined within a system of family relationships and social issues that have perpetuated them over the centuries.

Hence, there is a pressing need to work to enhance the relational impact of female cultural stereotypes in the medical field; this can improve treatment processes and deconstruct the male stereotypes responsible for poorer performance in the case of caregivers and lower treatment adherence in the case of patients. Patients must be made active and participatory subjects in sharing their treatment plans (Tsugawa et al., 2016).

## Building “counter narratives” for men

What appears to be more necessary than ever is to invest in projects and programs that bring men closer to the dimension of care (Ottaviano and Persico, 2020). In fact, we are too often oriented toward countering female stereotypes when, instead, we should realize that issues concerning women affect men, too.

What is needed are sexual and emotional education interventions for children and young individuals who live immersed in a social and media context that precedes and prepares them for entry into the peer group during adolescence. In this context, the threat of homophobia is the most powerful weapon for regulating male behavior within a very specific path, in which weaknesses, vulnerability, or even just the gentleness of gestures, postures, and words are accused of being effeminate.

Men—accustomed and socialized to dominating themselves and the world, autonomy, and responsibility—cannot afford to show vulnerability, limitations, or the need for help. “Hegemonic” masculinity does not foresee this, and proof of this is the fact that those who wish to experiment with different forms of masculinity find themselves caged by the coercive force of the traditional model and, at the same time, are disoriented by the lack of alternative reference paradigms.

It is therefore necessary to put counternarratives into circulation as a tool and methodology for deserting the plot, which significantly structures the reality we experience. The underlying theme of these narratives could be vulnerability, to be interpreted not as a condition of failure, pain, inferiority, or absence of strength, but as exposure of oneself to others, which leads to rethinking the more general concept of human: we are born exposed, delivered, vulnerable, by virtue of our very corporeality, which makes us dependent on those we do not yet know and in need of care. This happens well before the formation of the processes of subjectivation; vulnerability, in fact, precedes the formation of the “I” because we are born at the will of others, delivered to a world in which we exist by virtue of others (Ottaviano and Persico, 2020).

Therefore, “showing” one's vulnerability can become a precious resource for men to go beyond cultural imperatives and role stereotypes. This allows us to question the prevailing models of autonomy and self-sufficiency embodied by the traditional Western male subject. This does not mean essentializing the feminine as an archetype of vulnerability, nor, much less, considering it as helpless, resigned passivity, or as a value in itself. Nor does it mean thinking of women as potentially mothers who are “naturally” capable of caring practices. Rather, it means recovering the history of women's care as a heritage and wealth to be told and valued, sharing it with men. Care—understood as a concern for others—is a need at every stage of life. It is learned because care is transmitted, practiced, and refined through the experiences and narratives that give it shape.

## Social neuroscience as a new frontier for constructing a new counter-narrative

A particularly relevant research perspective for this topic is that of social neuroscience, particularly studies on empathic concern and clinical empathy (Decety et al., 2014; Eisenberg, 2013; Santiesteban, 2025). Within this theoretical framework, empathy is understood as a multidimensional construct that includes affective, cognitive, and motivational components, among which is the ability to understand the emotional state of others and the tendency to experience concern oriented toward others' well-being (Eisenberg, 2013; Christov-Moore et al., 2014). Empathic concern, in particular, represents an other-oriented emotional response that promotes prosocial and caregiving behaviors, proving crucial in healthcare contexts (Decety et al., 2014;Lown et al., 2011; Moudatsou et al., 2020).

In the context of the doctor–patient relationship, clinical empathy is not only configured as a relational competence but as a fundamental element for the quality of diagnosis and treatment adherence (Derksen et al., 2013; Hojat et al., 2023). It involves the physician's ability to understand the patient's subjective experience, communicate it effectively, and use it as a guide for therapeutic intervention (Hojat et al., 2023; Riess, 2017). Unlike a purely emotional response, clinical empathy requires a conscious regulation of one's own emotions, allowing the professional to maintain a balance between involvement and professional distance (Decety et al., 2014).

From this perspective, the link between empathy and gender representations assumes a central role and calls for broader critical reflection. Empathic competences, and particularly empathic concern, have historically and culturally been associated with femininity within a symbolic construction that has attributed to women the role of caregiving, attention to others, and management of relationships (Christov-Moore et al., 2014). This process of attribution, rooted in socialization models and normative systems, has, on the one hand, contributed to confining women to roles of lesser power and recognition, and, on the other hand, to fostering the development and valorization of specific relational and emotional skills.

The stereotype, therefore, is configured as an ambivalent structure: on the one hand, it is a limiting device that has historically justified inequalities and subordination; on the other, if critically reinterpreted, it is a potential resource, as far as it embodies competences developed over time through concrete social practices. In the clinical domain, this ambivalence becomes particularly evident. Empathic skills, although long considered “natural” and therefore devalued in terms of professional competence, are now revealed— in light of neuroscientific evidence— as fundamental elements for the effectiveness of the therapeutic relationship (Derksen et al., 2013; Riess, 2017; Moudatsou et al., 2020).

This reinterpretation allows for an important theoretical shift: it is not a matter of naturalizing empathy as an essential trait of femininity, but of recognizing how socialization processes have favored, to a greater extent in women, the exercise and refinement of these competences. In this sense, the stereotype can be transformed from a normative constraint into an epistemic and practical resource, capable of informing new models of healthcare professionalism. Empathic concern, from an element relegated to the private and affective sphere, thus becomes a central clinical competence, transferable and teachable, that can be integrated into training pathways regardless of gender (Batt-Rawden et al., 2013; Hojat et al., 2023).

In line with this perspective, the differences observed in communication styles between male and female physicians can be understood as the expression of greater social legitimization, for women, in the use of empathic and relationship-oriented modalities (Roter and Hall, 2004; Surchat et al., 2022). This highlights how gender norms not only constrain behaviors but also enable specific practices, making certain competencies more accessible and recognized within particular groups. The challenge, therefore, is not to eliminate these differences but to deconstruct their prescriptive character, making these competencies a shared asset.

The integration of contributions from social neuroscience and clinical empathy research thus makes it possible to construct a new counter-narrative that values the relational dimension of medicine, promoting professional models in which listening, understanding, and empathic response represent central and transversal competences, regardless of gender, while still recognizing the historical and cultural contribution through which these competences have been more extensively developed and attributed to femininity (Decety et al., 2014; Hojat et al., 2023).
